# Supplementary material for: Salmonella Typhimurium reprograms macrophage metabolism via T3SS effector SopE2 to promote intracellular replication and virulence
Source: Nat Commun. 2021 Feb 9;12:879. doi: 10.1038/s41467-021-21186-4 (PMC7873081; doi:10.1038/s41467-021-21186-4)
Supplement: Supplementary file 1 — Supplementary Information [file 41467_2021_21186_MOESM1_ESM.pdf]

Supplementary Information

Salmonella Typhimurium reprograms macrophage metabolism via T3SS effector SopE2 to promote intracellular replication and virulence

Lingyan Jiang[1,2,6], Peisheng Wang [1,2,6], Xiaorui Song [1,2,6], Huan Zhang [1,2], Shuangshuang Ma [1,2], Jingting Wang [1,2], Wanwu Li [1,2], Runxia Lv [1,2], Xiaoqian Liu [1,2], Shuai Ma [1,2], Jiaqi Yan[3], Haiyan Zhou[4], Di Huang[1,2], Zhihui Cheng[1,3], Chen Yang[4], Lu Feng [1,2,*], and Lei Wang [1,2,5,*]

[1] The Key Laboratory of Molecular Microbiology and Technology, Ministry of Education, Nankai University, Tianjin, China

[2] TEDA Institute of Biological Sciences and Biotechnology, Tianjin Key Laboratory of Microbial Functional Genomics, Nankai University, Tianjin, China

[3] College of Life Sciences, Nankai University, Tianjin, China

[4] CAS-Key Laboratory of Synthetic Biology, CAS Center for Excellence in Molecular Plant Sciences, Shanghai Institute of Plant Physiology and Ecology, Chinese Academy of Sciences, Shanghai, China

[5] The Institute of Translational Medicine Research, Tianjin Union Medical Center, Nankai University Affiliated Hospital, Nankai University, Tianjin, China

[6] These authors contributed equally: Lingyan Jiang, Peisheng Wang, Xiaorui Song.

[*] Correspondence: Lei Wang (e-mail: wanglei@nankai.edu.cn) and Lu Feng (e-mail: fenglu63@nankai.edu.cn)

- Supplementary Figures

- Supplementary Tables

- Supplementary Figures

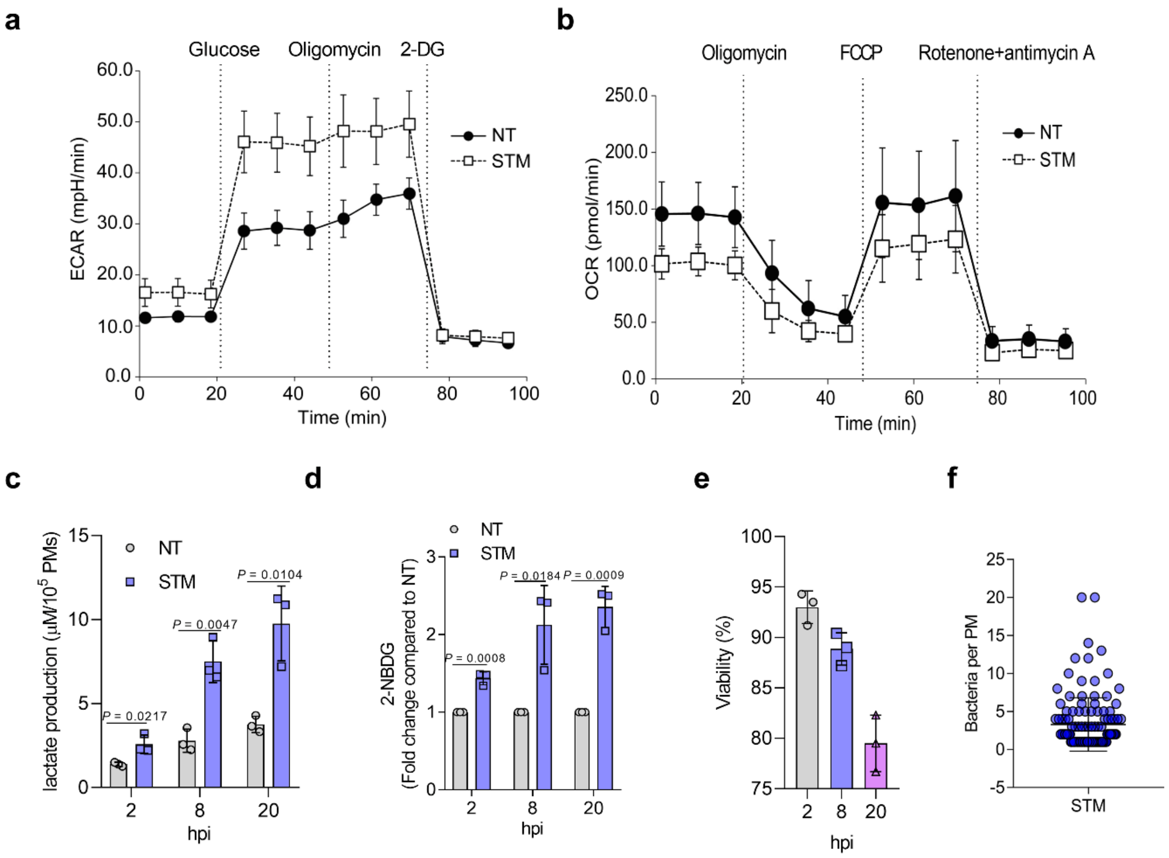

Supplementary Fig. 1 (related to Fig. 1) STM infection enhances glycolysis in infected macrophages. a,b, Real-time changes in the ECAR (a) and OCR (b) of peritoneal macrophages (PMs) left untreated (NT) or infected with STM for 8 h. Data are representative of three independent experiments and presented as mean ± SD. **c,** Lactate production by untreated PMs or those infected with STM for 2, 8, or 20 h. **d,** 2-NBDG uptake by untreated PMs or those infected with STM for 2, 8, or 20 h. **e,** Viability of PMs after infection with STM for 2, 8 and 20 h. The percentage of viable cells was determined by dividing the number of viable cells at 2, 8 and 20 h to that of 0 h. Data are presented as mean ± SD, n = 3 independent experiments (**c**–**e**). *P* values were determined using one-way ANOVA (**c, d**). **f,** Number of intracellular bacteria per PM. PMs were seeded on coverslips and infected with the wild-type (wt) STM strain (MOI = 10). Infected cells were fixed at 8 hours post-infection (hpi) and prepared for immunofluorescence staining. The number of intracellular bacteria per infected cell were

counted in random fields. Data are presented as mean ± SD, n = 126 cells examined over three independent experiment. Source data are included in Source Data file.

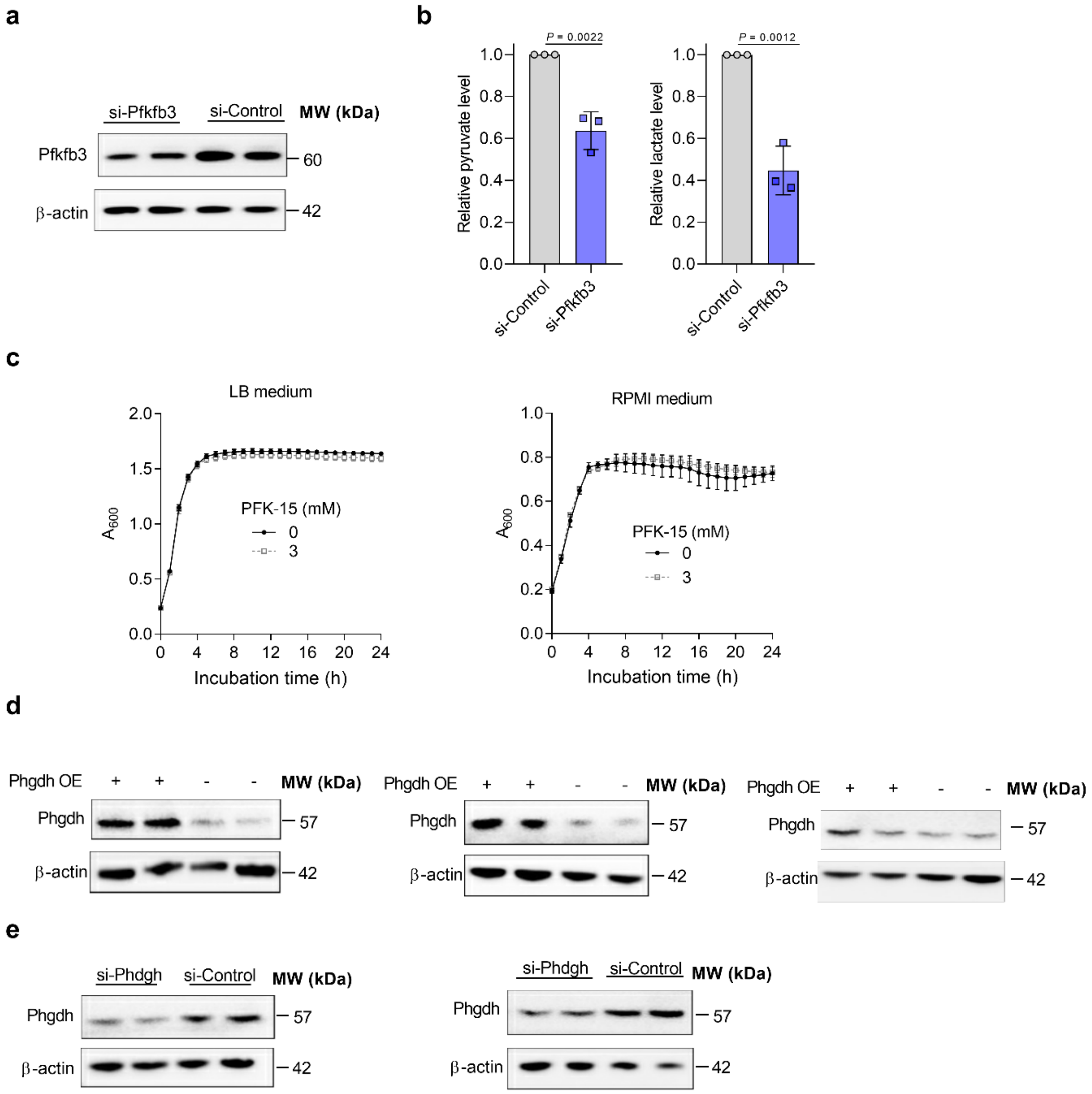

Supplementary Fig. 2 (related to Fig. 2) STM enhances glycolysis and reduces serine synthesis in macrophages to promote STM intracellular replication. a, Immunoblot analysis of Pfkfb3 protein levels in Pfkfb3 siRNA-treated or control siRNA-treated RAW264.7 cells. **b**, Pyruvate and lactate levels in Pfkfb3 siRNA-treated or control siRNA-treated RAW264.7 cells. **c,** Growth curves of STM in LB and RPMI medium in the presence or absence of 3 mM PFK-15. **d,** Immunoblot analysis of Phgdh protein levels in *phgdh*-overexpressing (OE: +) or control (OE: -) RAW264.7 cells. **e,** Immunoblot analysis of Phgdh protein levels in Phgdh siRNA-treated or control siRNA-treated RAW264.7 cells. Immunoblots are

representative of three independent experiments (**a, d, e**). Data are presented as mean ± SD, n = 3 independent experiments (**b, c**). *P* values were determined using two-tailed unpaired Student's *t*-test (**b**). Source data are included in Source Data file.

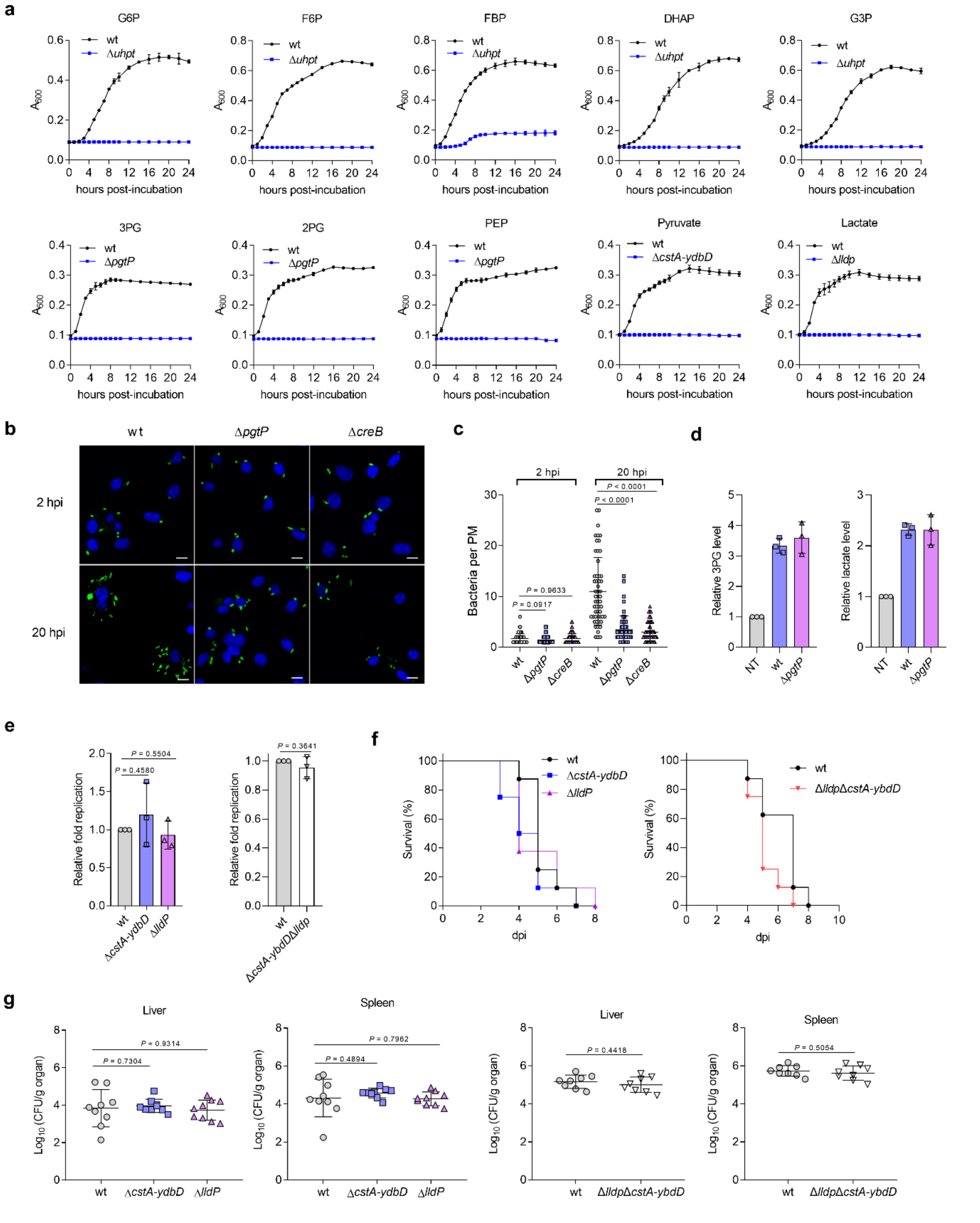

Supplementary Fig. 3 (related to Fig. 3) STM utilizes macrophage-derived 3PG as a major carbon source to support intracellular replication and systemic infection. a, Growth curves of wt STM and the indicated mutants in N-minimal medium containing 0.2% G6P, F6P, FBP, G3P, DHAP, 3PG, 2PG, PEP, pyruvate, or lactate as the sole carbon source. Data are presented

as mean ± SD, n = 3 independent experiments. **b,** Representative images of PMs infected with the wt, *pgtP* mutant, or *creB* mutant STM strain at 2 and 20 hpi. Green, STM; blue, nuclei. Scale bars, 10 μm. PMs were seeded on coverslips and infected with wt STM, *pgtP* mutant, or *creB* mutant (MOI = 10). Infected cells were fixed at 2 and 20 hpi and prepared for immunofluorescence staining. **c,** Intracellular bacteria per cell were counted in random fields from Z-stack images. The bars indicate the mean number of bacteria contained in the infected cells that were counted. Data are presented as mean ± SD. 2 hpi: n = 88, 64, and 86 cells examined for wt, *pgtP* mutant, and *creB* mutant respectively over three independent experiment; 20 hpi: n = 55, 57, and 63 cells examined for wt, *pgtP* mutant, and *creB* mutant respectively over three independent experiment. **d**, 3PG and lactate levels in PMs infected with wt STM or *pgtP* mutant for 8 h. Data are presented as mean ± SD, n = 3 independent experiments. **e,** Replication assays of wt STM, *cstA-ydbD* mutant, *lldP* mutant, and *cstA-ybdD lldP* double mutant in PMs. Data are presented as mean ± SD, n = 3 independent experiments. **f,** Survival curves of mice infected i.p. with wt STM, *cstA-ydbD* mutant, *lldP* mutant, or *cstA-ybdD lldP* double mutant. n = 8 mice per group. **g,** Liver and spleen bacterial burdens in mice infected with wt STM, *cstA-ydbD* mutant, *lldP* mutant, or *cstA-ybdD lldP* double mutant. Data are presented as mean ± SD. Left: n = 9 mice per group; right: n = 8 mice per group. *P* values were determined using two-tailed unpaired Student's *t*-test (**c, d, e**), log-rank Mantel-Cox test (**f**) or Mann-Whitney U test (**g**). Source data are included in Source Data file.

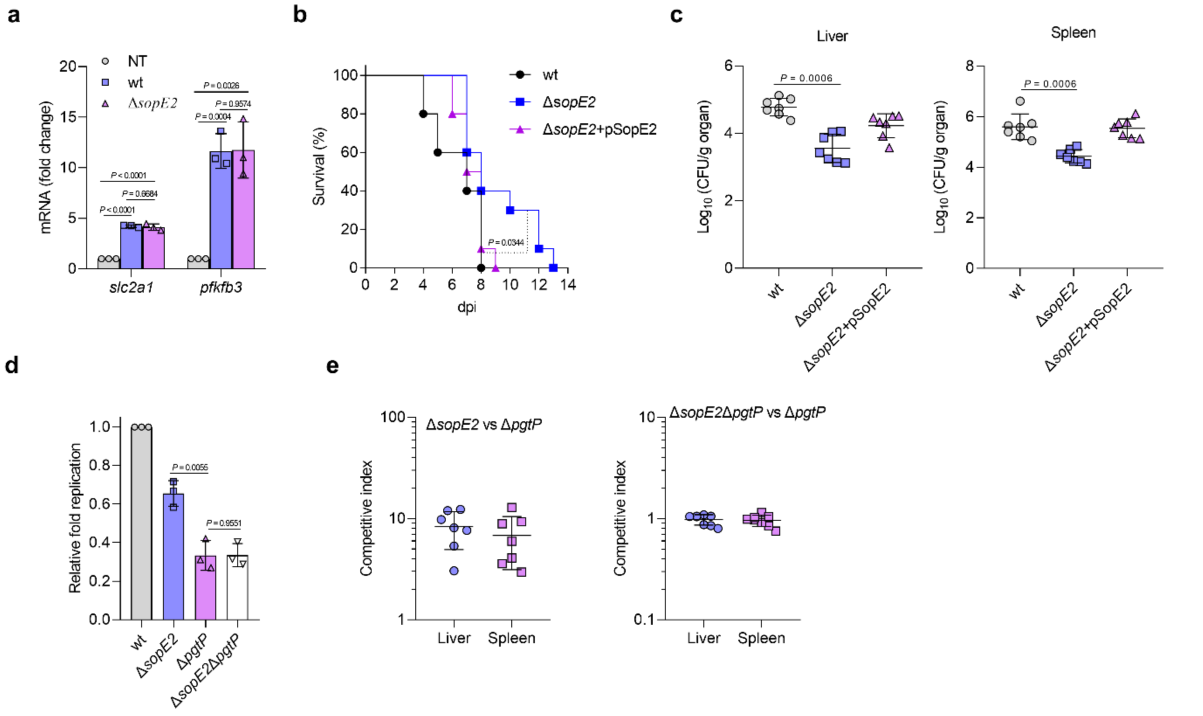

Supplementary Fig. 4 (related to Fig. 4) STM uses the SPI-1 effector SopE2 to repress macrophage serine synthesis. a, RT-qPCR analysis of *slc2a1* and *pfkfb3* mRNA levels in untreated (NT) PMs or PMs infected with wt STM, or *sopE2* mutant for 8 h. **b,** Survival curves of mice infected i.p. with the wt, *sopE2* mutant, or complemented strain (n = 10 mice per group). **c,** Liver and spleen bacterial burdens in mice infected with the wt, *sopE2* mutant, or complemented strain. **d**, Replication assays of wt STM, *sopE2* mutant, *sopE2* complemented strain, and *sopE2 pgtP* double mutant in PMs. **e**, Competitive index of the *sopE2* mutant and the *sopE2 pgtP* double mutant versus the *pgtP* mutant in the liver and spleen of infected mice. Data are presented as mean ± SD, n = 3 independent experiments (**a, d**), n = 7 mice per group (**c, e**). *P* values were determined using one-way ANOVA (**a**), log-rank Mantel-Cox test (**b**), Mann-Whitney U test (**c, e**), or two-tailed unpaired Student's *t*-test (**d**). *P* = 0.0008 and 0.0006 for liver and spleen respectively based on raw CFU values of the *sopE2* mutant vs. *pgtP* mutant; *P* = 0.9591 and 0.8785 for liver and spleen respectively based on raw CFU values of the *sopE2 pgtP* double mutant vs. *pgtP* mutant (**e**). Source data are included in Source Data file.

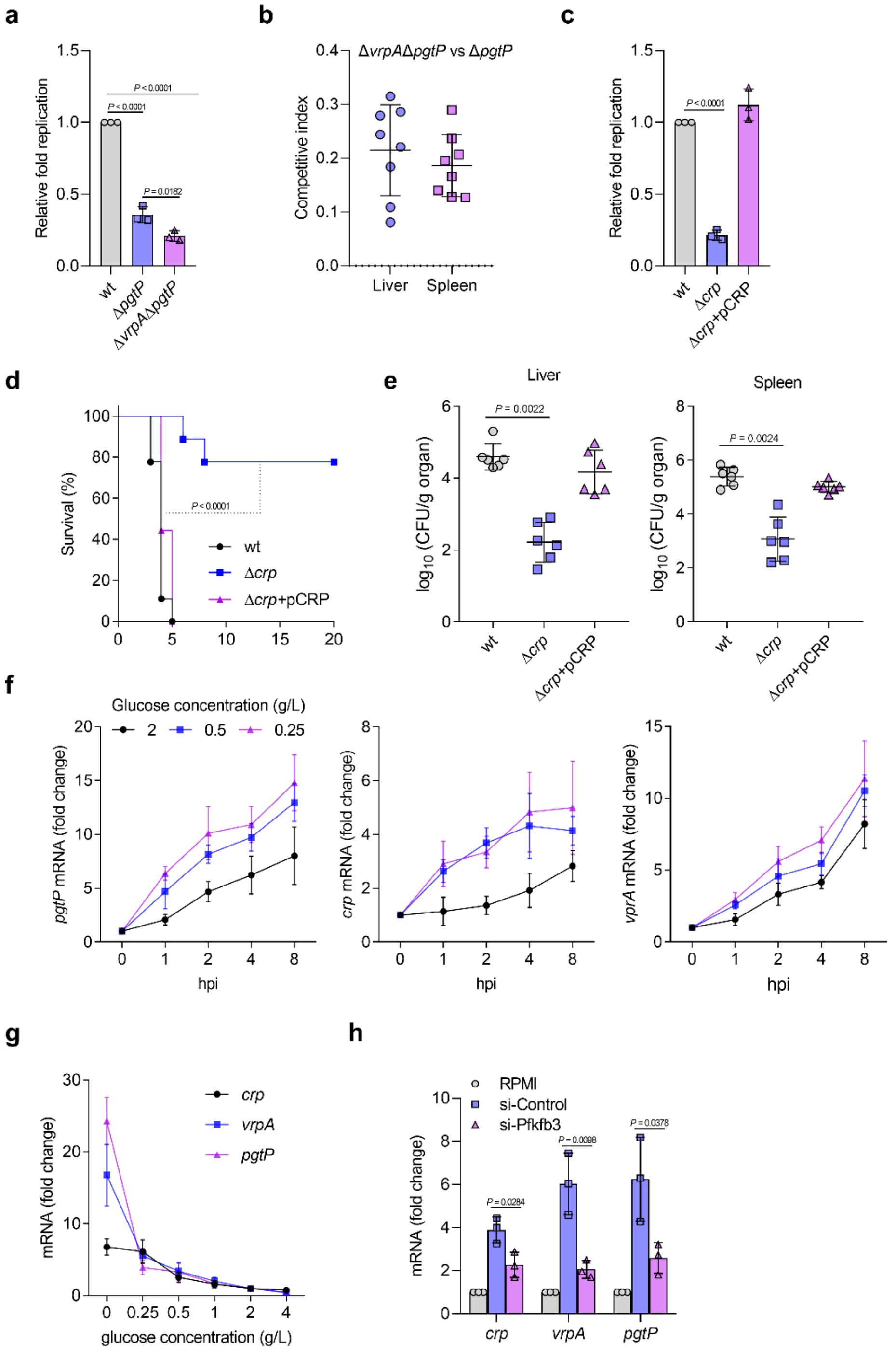

Supplementary Fig. 5 (related to Fig. 5) STM senses decreased glucose levels in macrophages to upregulate bacterial 3PG uptake. a, Replication of the STM wt, *pgtP* mutant, and *vrpA pgtP* double mutant in PMs. **b,** Competitive index of the *vrpA pgtP* double mutant versus the *pgtP* mutant in the liver and spleen of infected mice. **c,** Replication of the wt, *crp* mutant, and *crp* complemented strains in PMs. **d,** Survival curves of mice infected i.p. with

the wt, *crp* mutant, or *crp* complemented strain. n = 9 mice per group. **e,** Liver and spleen bacterial burdens in mice infected with the wt, *crp* mutant, or *crp* complemented strain. **f,** RT-qPCR analysis of *crp*, *vrpA*, and *pgtP* mRNA levels in PMs cultured in RPMI medium containing different glucose concentrations. PMs used for infection were cultured in RPMI medium containing 2, 0.5, or 0.25 g/L glucose and infected with the wt STM strain at an MOI of 10. Fold changes in *crp*, *vrpA* and *pgtP* intracellular expression levels at 1, 2, 4, and 8 hpi relative to the expression levels of the corresponding genes in RPMI medium (0 hpi) are presented. **g,** RT-qPCR analysis of *crp*, *vrpA*, and *pgtP* gene expression in wt STM cultured in RPMI medium containing different glucose concentrations. wt STM was grown in RPMI medium containing 0, 0.25, 0.5, 1, 2, or 4 g/L glucose for 6 h before collection. Fold changes in gene expression in bacteria cultured in RPMI containing 0, 0.25, 0.5, 1, or 4 g/L glucose relative to the expression levels of the corresponding genes in bacteria cultured in RPMI containing 2 g/L glucose are presented. **h**, RT-qPCR analysis of STM *crp*, *vrpA*, and *pgtP* mRNA levels at 8 h infection of control siRNA-treated and Pfkfb3 siRNA-treated RAW264.7 cells, relative to those in RPMI medium. Data are presented as mean ± SD, n = 3 independent experiments (**a, c, f–h),** n = 8 (**b**) or n = 6 (**e**) mice per group**.** *P* values were determined using two-tailed unpaired Student's *t*-test (**a, c**), Mann-Whitney U test (**b, e**), log-rank Mantel-Cox test (**d**) or one-way ANOVA (**h**). *P* = 0.0006 and 0.0002 for liver and spleen respectively based on raw CFU values of the *vrpB pgtP* double mutant vs. *pgtP* mutant (**b**). Source data are included in Source Data file.

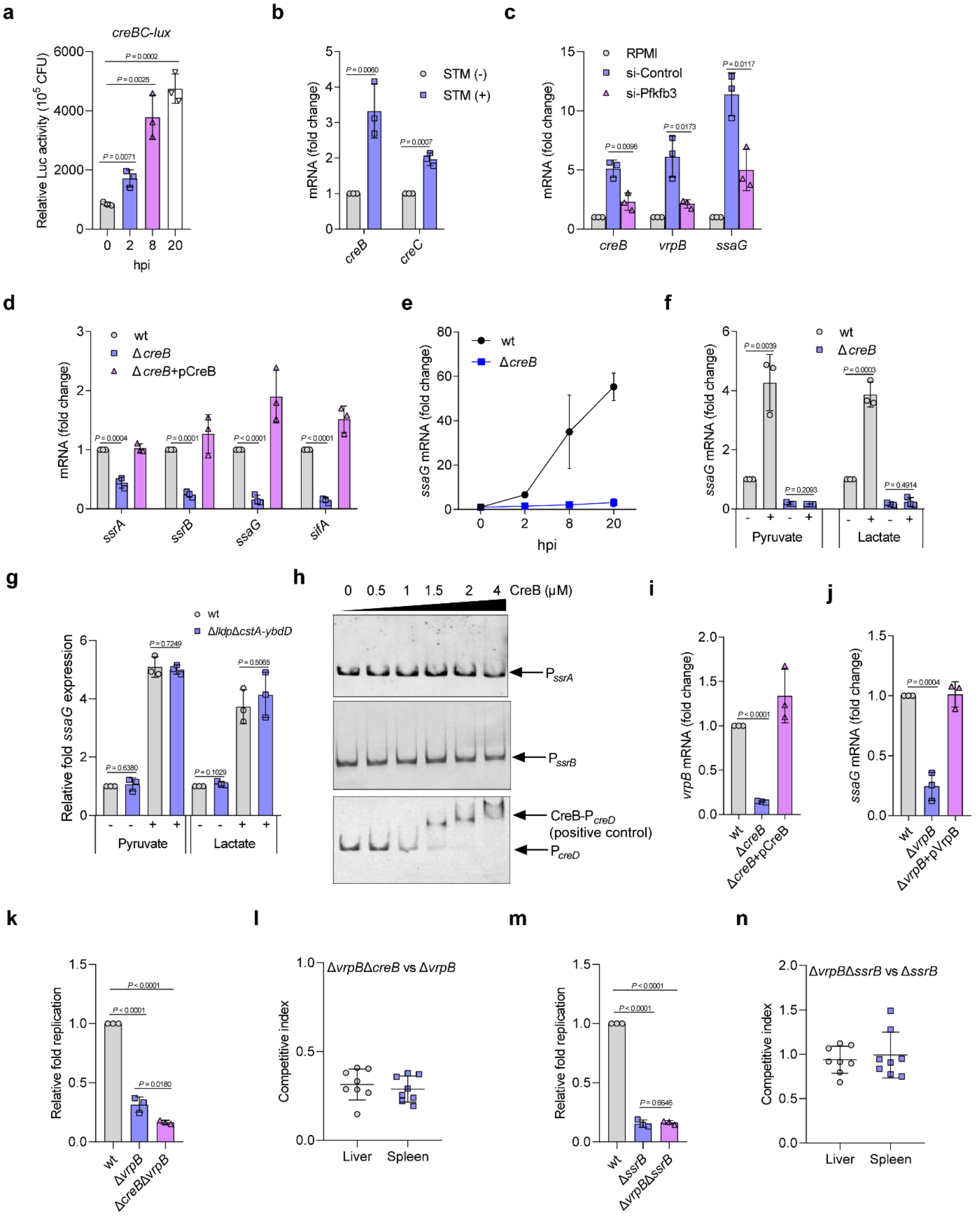

Supplementary Fig. 6 (related to Fig. 6) CreBC responds to increased macrophage pyruvate and lactate levels to activate SPI-2 genes through VrpB. a, Expression of the *creBC-lux* transcriptional fusions was analysed in the STM wt growth in RPMI medium (0 h) and inside PMs at 2, 8, and 20 h. **b**, RT-qPCR analysis of *creB* and *creC* mRNA levels in STM

wt cultured with lysate from STM-infected PMs (STM+) or lysate from uninfected PMs (STM-). **c,** RT-qPCR analysis of STM *creB*, *vrpB*, and *ssaG* mRNA levels at 8 h infection of control siRNA-treated and Pfkfb3 siRNA-treated RAW264.7 cells, relative to those in RPMI medium. **d**, RT-qPCR analysis of *ssrA*, *ssrB*, *ssaG*, and *sifA* mRNA levels in the wt, *creB* mutant, and *creB*-complemented strains. Bacteria were grown in N-minimal medium for 6 h before collection. **e,** RT-qPCR analysis of *ssaG* mRNA levels in intracellular wt and *creB* mutant. PMs were infected with wt or *creB* mutant at an MOI of 10. Fold changes in *ssaG* intracellular expression at 2, 8, and 20 hpi relative to its expression in RPMI medium (0 hpi) are presented. **f,** RT-qPCR analysis of wt or *creB* mutant cultured in N-minimal medium in the presence or absence of 1 mM pyruvate or lactate. Fold changes in *ssaG* expression in the mutant relative to that in the wt strain are presented. **g,** RT-qPCR analysis of wt or *cstA-ybdD lldP* double mutant cultured in N-minimal medium in the presence or absence of 1 mM pyruvate or lactate. Fold changes in *ssaG* expression in the mutant relative to that in the wt strain are presented. **h,** EMSA of *ssrA* and *ssrB* promoter DNA fragments with purified CreB protein. **i,** RT-qPCR analysis of *vrpB* mRNA levels in the wt, *creB* mutant, and *creB* complemented strains. Bacteria were grown in N-minimal medium for 6 h before collection. **j,** RT-qPCR analysis of *ssaG* mRNA levels in the wt, *vrpB* mutant, and *vrpB* complemented strains. Bacteria were grown in N-minimal medium for 6 h before collection. **k**, Replication of the wt, *vrpB* mutant, and *creB vrpB* double mutant in PMs. **l**, Competitive index of the *creB vrpB* double mutant versus the *vrpB* mutant in the liver and spleen of infected mice. **m**, Replication of the wt, *ssrB* mutant, and *vrpB ssrB* double mutant in PMs. **n**, Competitive index of the *vrpB ssrB* double mutant versus the *ssrB* mutant in the liver and spleen of infected mice. Data are presented as mean ± SD, n = 3 independent experiments (**a–g, i–k**, **m),** n = 8 mice per group (**l, n)**. Images are representative of three independent experiments (**h**). *P* values were determined using two-tailed unpaired Student's *t*-test (**a, f, g, i–k**, **m**), one-way ANOVA (**b–d**) or Mann-Whitney U test (**l, n)**. *P* = 0.0224 and 0.0207 for liver and spleen respectively based on raw CFU values of the *vrpB creB* double mutant vs. *creB* mutant (**l**). *P* = 0.9591 and 0.9591 for liver and spleen respectively

based on raw CFU values of the *vrpB ssrB* double mutant vs. *ssrB* mutant (**l**). Source data are included in Source Data file.

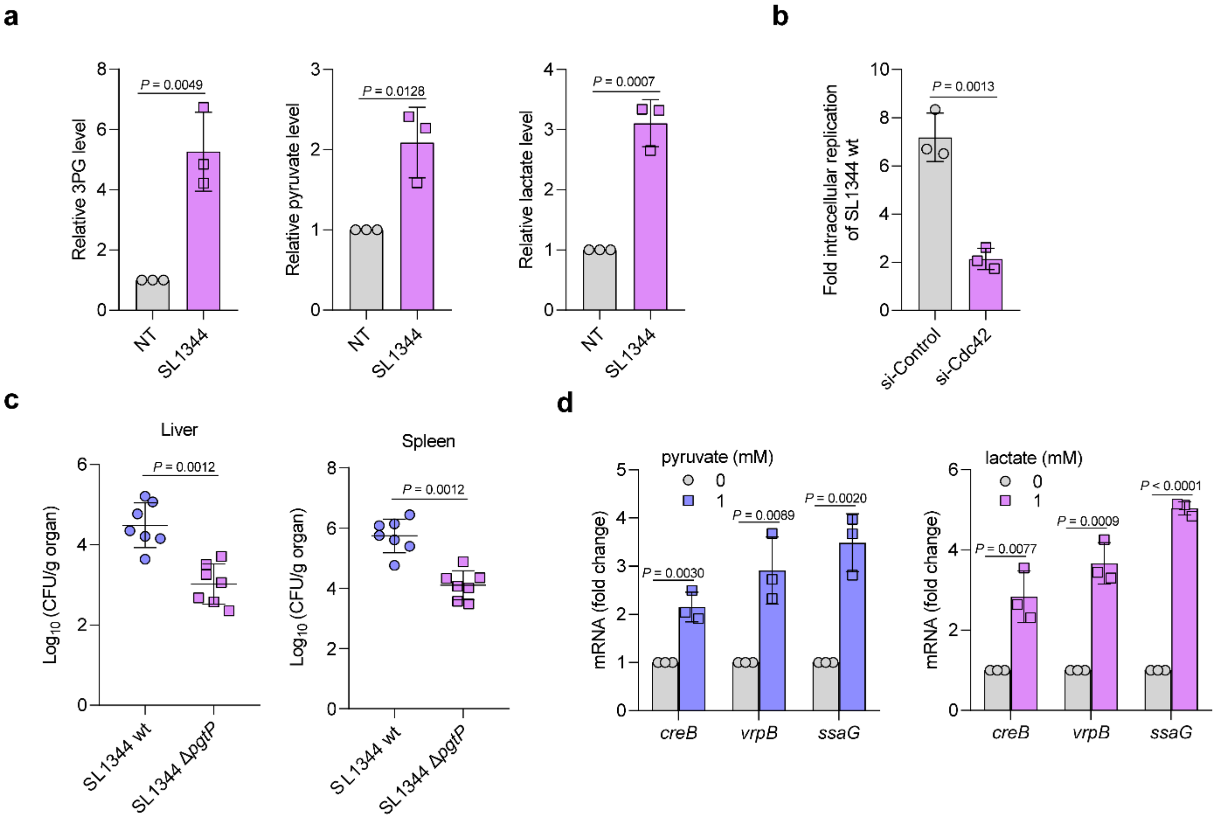

Supplementary Fig. 7 STM SL1344 strain induces the same metabolic changes in macrophages and responses to those changes in the same way as 14028s does. **a,** 3PG, pyruvate and lactate levels by untreated PMs or those infected with STM SL1344 wt for 8 h. **b,** Replication of SL1344 wt in Cdc42 siRNA-treated or control siRNA-treated HeLa cells. **c,** Liver and spleen bacterial burdens in mice infected with the SL1344 wt or *pgtP* mutant. **d,** RT-qPCR analysis of *creB*, *vrpB*, and *ssaG* mRNA levels in SL1344 in the presence or absence of 1 mM pyruvate or lactate. Data are presented as mean ± SD, n = 3 independent experiments (**a, b, d**), n = 7 mice per group (**c**). *P* values were determined using two-tailed unpaired Student's *t*-test (**a, b**), Mann-Whitney U test (**c**), or one-way ANOVA (**d**).

- Supplementary Tables

Supplementary Table 1. Intracellular metabolite levels in infected and uninfected PMs (absolute quantification)

Metabolite	Concentration (*x* ×M/PM cell)			Fold (STM 8 h/NT)	*P* value	Concentration (*x* ×M/STM cell)	
	x	NT	STM 8 h			*x*	STM
hexose 6-phosphate (G6P/F6P)	10^{-13}	6.53±0.54	16.4±0.94	2.51	0.0003	10^{-16}	8.90±1.65
fructose 1,6-bisphosphate (FBP)	10^{-13}	9.07±0.91	34.5±3.41	3.80	0.0134	10^{-16}	4.76±0.77
dihydroxyacetone phosphate (DHAP)	10^{-13}	5.38±0.67	26.5±6.50	4.93	0.0089	10^{-16}	3.54±1.10
phosphoglycerate (2PG/3PG)	10^{-13}	3.20±0.64	25.2±0.37	7.88	0.0020	10^{-16}	2.95±0.85
phosphoenolpyruvate (PEP)	10^{-13}	2.51±0.07	12.3±1.24	4.90	0.0004	10^{-16}	8.63±1.43
pyruvate	10^{-12}	5.19±0.29	9.78±0.79	1.88	0.0043	10^{-15}	1.44±0.51
lactate	10^{-11}	6.61±1.67	17.4±1.44	2.63	0.0035	10^{-14}	7.98±2.23
citrate (Cit)	10^{-12}	4.93±0.74	2.03±0.33	0.41	1.8851E-08	10^{-15}	2.01±0.40
α-ketoglutarate (α-KG)	10^{-12}	6.38±0.14	10.2±1.41	1.60	0.0059	10^{-15}	2.64±0.21
succinate (Succ)	10^{-11}	1.47±0.38	6.71±1.80	4.56	0.0238	10^{-14}	1.50±0.29
fumarate (Fum)	10^{-11}	3.33±0.22	4.21±0.13	1.26	0.0048	10^{-14}	1.20±0.22
malate (Mal)	10^{-11}	6.18±0.22	2.91±0.19	0.47	3.6706E-05	10^{-14}	8.06±2.63
oxaloacetate (OAA)	10^{-13}	7.42±0.84	7.43±1.28	1.00	0.9896	10^{-16}	2.27±0.47
serine	10^{-12}	80.7±2.51	18.3±2.62	0.23	1.7E-08	10^{-15}	1.44±0.78
glycine	10^{-11}	17.1±1.32	2.19±0.05	0.13	2.704E-10	10^{-14}	3.46±0.84
glutathione (GSH)	10^{-13}	14.2±2.83	6.48±1.56	0.46	0.0002	10^{-16}	1.52±0.38
6-phosphogluconate (6PG)	10^{-13}	2.50±0.19	2.07±0.58	0.83	0.2868	10^{-16}	2.06±0.51
ribose 5-phosphate (R5P)	10^{-12}	1.57±0.37	1.85±0.24	1.18	0.3925	10^{-16}	6.49±1.34
erythrose 4-phosphate (E4P)	10^{-13}	6.11±0.58	5.86±1.18	0.96	0.5948	10^{-16}	1.13±0.44

Data are presented as mean ± SD, n = 3 independent experiments. *P* values were determined using one-way ANOVA. NT, untreated peritoneal macrophages (PMs); STM 8 h, PMs infected for 8 h with *Salmonella enterica* serovar Typhimurium (STM).

Supplementary Table 2. Intracellular metabolite levels in infected and uninfected PMs (relative quantification)

**Metabolite**	**NT**	**Live STM**	**Heat-killed STM**	**Fold change (Live STM/NT)**	***P* value**	**Fold change (Heat-killed STM/NT)**	***P* value**
hexose 6-phosphate (G6P/F6P)	261±20	662±130	562±99	2.54	0.0061	2.16	0.0067
fructose 1,6-bisphosphate (FBP)	2867±112	12346±221	7886±661	4.31	3.11E-07	2.75	0.0002
dihydroxyacetone phosphate (DHAP)	342±18	1467±152	1404±224	4.29	0.0002	4.10	2.76E-05
phosphoglycerate (2PG/3PG)	26±6	127±4	49±9	4.84	1.69E-05	1.86	0.0231
phosphoenolpyruvate (PEP)	283±38	1493±81	2434±242	5.28	1.98E-05	8.61	0.0001
pyruvate	53±5	105±9	61±15	1.98	0.0011	1.14	0.44
lactate	501±26	2142±212	1830±195	4.27	0.0002	3.65	0.0003
serine	25±1	15±2	71±21	0.61	0.0033	2.85	0.0218
glycine	27±2	14±7	66±21	0.54	0.0566	2.48	0.0333
glutathione (GSH)	123±10	77±8	245±27	0.63	0.0037	1.99	0.0017

Data are presented as mean ± SD, n = 3 independent experiments. *P* values were determined using one-way ANOVA. NT, untreated peritoneal macrophages (PMs); Live STM, PMs infected for 8 h with live *Salmonella enterica* serovar Typhimurium (STM); Heat-killed STM, PMs infected for 8 h with heat-killed STM.

Supplementary Table 3. List of the putative regulatory proteins that present only in STM but not in *Escherichia coli*

Gene	Function
STM14_0037	[protein=putative transcriptional regulator] [protein_id=ACY86574.1]
STM14_0039	[protein=putative transcriptional regulator] [protein_id=ACY86576.1]
STM14_0401	[protein=putative response regulator] [protein_id=ACY86921.1]
STM14_0405	[protein=putative response regulator] [protein_id=ACY86925.1]
STM14_0485	[protein=putative regulatory protein] [protein_id=ACY87005.1]
STM14_0642	[protein=putative regulatory protein] [protein_id=ACY87159.1]
STM14_0676	[protein=putative regulatory protein] [protein_id=ACY87188.1]
STM14_1429	[protein=putative regulator] [protein_id=ACY87915.1]
STM14_1526	[protein=putative response regulator] [protein_id=ACY88005.1]
STM14_1866	[protein=putative transcriptional regulator] [protein_id=ACY88338.1]
STM14_1901	[protein=putative transcriptional regulator] [protein_id=ACY88372.1]
STM14_2708	[protein=putative transcriptional regulator] [protein_id=ACY89151.1]
STM14_3217	[gene=STM14_3217] [protein=putative regulator] [protein_id=ACY89646.1]
STM14_3313 (VrpA)	[protein=putative transcriptional regulator] [protein_id=ACY89735.1]
STM14_3563	[protein=putative transcriptional regulator] [protein_id=ACY89976.1]
STM14_3742	[protein=putative transcriptional regulator] [protein_id=ACY90148.1]
STM14_4848	[protein=putative regulatory protein] [protein_id=ACY91205.1]
STM14_5217	[protein=putative regulatory protein] [protein_id=ACY91555.1]

Supplementary Table 4. Strains and plasmids used in this study

Plasmid or strain	Genotype or description	Source
**Plasmids**		
pKD46	λ-Red recombinase system under an arabinose-inducible promoter; Ap^R	Laboratory collection
pKD3	template plasmid containing the Cm cassette for λ-Red recombination; Cm^R	Laboratory collection
pKD4	template plasmid containing the Km cassette for λ-Red recombination; Km^R	Laboratory collection
pCP20	temperature-sensitive replicon expressing the FLP gene to abolish the antibiotic resistance of the mutant strains; Ap^R	Laboratory collection
pET28a	T_7 expression vector; Km^R	Laboratory collection
pWSK129	low-copy-number expression vector; Km^R	Laboratory collection
pWSK-3×FLAG	pWSK129 carrying a 3×FLAG sequence and the Cm cassette; Cm^R, Km^R	Laboratory construction
pPgtP	pWSK129 carrying the wt *pgtP* gene; Km^R	This study
pSopE2	pWSK129 carrying the wt *sopE2* gene; Km^R	This study
pCreB	pWSK129 carrying the wt *creB* gene; Km^R	This study
pVrpA	pWSK129 carrying the wt *vrpA* gene; Km^R	This study
pVrpB	pWSK129 carrying the wt *vrpB* gene; Km^R	This study
pCRP	pWSK129 carrying the wt *crp* gene; Km^R	This study
pSsrB	pWSK129 carrying the wt *ssrB* gene; Km^R	[1]
pET28a-CreB	pET28a carrying the STM *creB* gene; Km^R	This study
pET28a-Crp	pET28a carrying the STM *crp* gene; Km^R	This study
pET28a-VrpA	pET28a carrying the STM *vrpA* gene; Km^R	This study
pET28a-VrpB	pET28a carrying the STM *vrpB* gene; Km^R	This study
creBC-lux	pMS402 carring carrying the STM *creBC* promoter; Km^R	This study
**Bacterial strains**		
Escherichia coli (BL21)	F^- *ompT* $hsdS_B$ with a prophage carrying the T_7 RNA polymerase	ATCC
wild-type (wt) STM	wild-type *Salmonella* enterica serovar Typhimurium strain ATCC 14028s	Laboratory collection
Δ*pgtP*	wt strain *pgtP*::Cm; Cm^R	This study
Δ*creB*	wt strain *creB*::Cm; Cm^R	This study

Δ*vrpA*	wt strain *vrpA*::Cm; Cm^{R}	This study
Δ*vrpB*	wt strain *vrpB*::Cm; Cm^{R}	This study
Δ*crp*	wt strain *crp*::Cm; Cm^{R}	This study
Δ*uhpT*	wt strain *uhpT*::Cm; Cm^{R}	This study
Δ*cstA-ydbD*	wt strain *cstA-ydbD*::Cm; Cm^{R}	This study
Δ*lldP*	wt strain *lldP*::Cm; Cm^{R}	This study
Δ*ptsG*/*manXYZ*/*glk*	wt strain *ptsG*, *manXYZ* and *glk* mutation; Cm^{R}	This study
ΔSPI-1	wt strain SPI-1::Cm; Cm^{R}	[2]
Δ*ssrB*	wt strain *ssrB*::Cm; Cm^{R}	[1]
Δ*sopE2*	wt strain *sopE2*::Cm; Cm^{R}	This study
Δ*steA*	wt strain *steA*::Cm; Cm^{R}	This study
Δ*steB*	wt strain *steB*::Cm; Cm^{R}	This study
Δ*sspH1*	wt strain *sspH1*::Cm; Cm^{R}	This study
Δ*gtgE*	wt strain *gtgE*::Cm; Cm^{R}	This study
Δ*sipABCD*	wt strain *sipABCD*::Cm; Cm^{R}	This study
Δ*slrP*	wt strain *slrP*::Cm; Cm^{R}	This study
Δ*sptP*	wt strain *sptP*::Cm; Cm^{R}	This study
Δ*sopA*	wt strain *sopA*::Cm; Cm^{R}	This study
Δ*sopB*	wt strain *sopB*::Cm; Cm^{R}	This study
Δ*sopD*	wt strain *sopD*::Cm; Cm^{R}	This study
Δ*avrA*	wt strain *avrA*::Cm; Cm^{R}	This study
Δ*lldP*Δ*cstA-ydbD*	wt strain *lldP*::Cm and *cstA-ydbD*::Km; Cm^{R}, Km^{R}	This study
Δ*sopE2*Δ*pgtP*	wt strain *sopE2*::Cm and *pgtP*::Km; Cm^{R}, Km^{R}	This study
Δ*vrpB*Δ*ssrB*	wt strain *vrpB*::Cm and *ssrB*::Km; Cm^{R}, Km^{R}	This study
Δ*creB*Δ*vrpB*	wt strain *vrpB*::Cm and *creB*::Km; Cm^{R}, Km^{R}	This study
Δ*pgtP*+pPgtP	Δ*pgtP* containing plasmid pPgtP; Cm^{R}, Km^{R}	This study
Δ*sopE2*+pSopE2	Δ*sopE2* containing plasmid pSopE2; Cm^{R}, Km^{R}	This study
Δ*vrpA*+pVrpA	Δ*vrpA* containing plasmid pVrpA; Cm^{R}, Km^{R}	This study
Δ*vrpA*+pPgtP	Δ*vrpA* containing plasmid pPgtP; Cm^{R}, Km^{R}	This study
Δ*crp*+pCRP	Δ*crp* containing plasmid pCRP; Cm^{R}, Km^{R}	This study
Δ*creB*+pCreB	Δ*creB* containing plasmid pCreB; Cm^{R}, Km^{R}	This study
Δ*vrpB*+pVrpB	Δ*vrpB* containing plasmid pVrpB; Cm^{R}, Km^{R}	This study
Δ*vrpB*+pSsrB	Δ*vrpB* containing plasmid pSsrB; Cm^{R}, Km^{R}	This study

wt+ *creBC-lux*	wt strain containing plasmid *creBC-lux*; Km^R	This study
CreB-FLAG	wt strain with the *creB* gene tagged with a 3×FLAG tag; Cm^R	This study
VrpA-FLAG	wt strain with the *vrpA* gene tagged with a 3×FLAG tag; Cm^R	This study
VrpB-FLAG	wt strain with the *vrpB* gene tagged with a 3×FLAG tag; Cm^R	This study
SL1344 wt	wild-type *Salmonella* enterica serovar Typhimurium strain SL1344	Laboratory collection
SL1344 Δ*pgtP*	SL1344 wt strain *pgtP*::Cm; Cm^R	This study
BL21+pET28a-CreB	BL21 containing the plasmid pET28a-CreB; Km^R	This study
BL21+pET28a-Crp	BL21 containing the plasmid pET28a-Crp; Km^R	This study
BL21+pET28a-VrpA	BL21 containing the plasmid pET28a-VrpA; Km^R	This study
BL21+pET28a-VrpB	BL21 containing the plasmid pET28a-VrpB; Km^R	This study

Supplementary Table 5. Oligonucleotides used in this study

Target gene		Primer sequence (5'–3')
Primers used for construction of the mutants*		
pgtP	F	TCAGAATTTATAATGCTCTTTACTTAGACCATATTTTT GTGTAGGCTGGAGCTGCTTCG
	R	TTATTGCGCGTCCGCAAGCTGCAGTGAGTCCTGATTAT CATATGAATATCCTCCTTAG
creB	F	ATGCAGCAACCGCAGGTCTGGTTAGTCGAGGATGAACA GTGTAGGCTGGAGCTGCTTCG
	R	TCAGACGCTCCTCAGGCTGTATCCCATTCCACGATGCG CATATGAATATCCTCCTTAG
vrpA	F	GTAATCAAAATTCAAGTAATACATGAATAGGTAAGGGA GTGTAGGCTGGAGCTGCTTCG
	R	ATAAAAACACGAGTGGATGAACAATCAATGCAAATAACC CATATGAATATCCTCCTTAG
vrpB	F	ATGGCGAACTGGGCGCAGAAATTGAAATTACATCACCT GTGTAGGCTGGAGCTGCTTCG
	R	CTAAACCTGGGCTAAAAAATACAAAAAGCGACTCAGGG CATATGAATATCCTCCTTAG
crp	F	TGGTGCTTGGCAAACCGCAAACAGACCCGACTCTTGAA GTGTAGGCTGGAGCTGCTTCG
	R	TTAACGGGTGCCGTAGACGACGATGGTCTTGCCATGCGC CATATGAATATCCTCCTTAG
cstA-ydbD	F	ATGAATAAATCAGGGAAATACCTCGTCTGGACAGCGCT GTGTAGGCTGGAGCTGCTTCG
	R	TCAGCAGCAACGCGCGCCGCCTTTTCCACCATAGCGCG CATATGAATATCCTCCTTAG
lldP	F	ATGAACCTCTGGCAACAAAACTATGACCCGGCGGGTAA GTGTAGGCTGGAGCTGCTTCG
	R	TCACGGAATCATCCAGGTTAAAACATAGGCCTGAAGCG CATATGAATATCCTCCTTAG
uhpT	F	TGCCATTCGCAGGTATAAAAATTAGCTCAGGAGTAATCC GTGTAGGCTGGAGCTGCTTC
	R	CGGCGTTAAGCCGGGCAAAACGTTACCAAATGCACACAT CATATGAATATCCTCCTTAG

gtgE	F	TAATTACATTAACAAAATTACTATTCGGCGAGTATATT GTGTAGGCTGGAGCTGCTTC
	R	GCGGTAGCCTGAATAATTATCTTGGTAAAGGTTAACTA CATATGAATATCCTCCTTAG
sipABCD	F	ATCTTTCCCGGTTAATTAACGCTGCATGTGCAAGCCAT GTGTAGGCTGGAGCTGCTTC
	R	CACAGTGAACAAGAAAAGGAATAATTATGGTAAATGAC CATATGAATATCCTCCTTAG
sopE2	F	AGTGTAGCTATGCATAGTTATCTAAAAGGAGAACTACC GTGTAGGCTGGAGCTGCTTC
	R	AATTCATATGGTTAATAGCAGTATTGTATTTACTACCA CATATGAATATCCTCCTTAG
slrP	F	TGTTACTTTAGGTTACGTTCAGATCAGGTAGGGAAAAT GTGTAGGCTGGAGCTGCTTC
	R	AAACAGGCTCTCTCCCTCTTCTGATAAACTGCGTTCAG CATATGAATATCCTCCTTAG
sspH1	F	TCTTCTATACTTTCAGTCTGACCGACTGGAGGTTTCAT GTGTAGGCTGGAGCTGCTTC
	R	ACCTCGGTATACGACCAGATTTTCCGATCTGCCAATAG CATATGAATATCCTCCTTAG
steA	F	CATATAAAGCTATTGAGCAAAATTTGAAGGAGTAGGAT GTGTAGGCTGGAGCTGCTTC
	R	TCTGATTTCTAACAAAACTGGCTAAACATAAACGCTTT CATATGAATATCCTCCTTAG
steB	F	ATTATTGTTAGTTTGAAATCAATCTCAGGTAATAATCC GTGTAGGCTGGAGCTGCTTC
	R	GTGGAATAGCAATGCCGGGAAGGACATGGCATGACACT CATATGAATATCCTCCTTAG
sopB	F	TGAGTCATTTGTGAATCAGCAGGAAGCGCTCAAAAACATACTGCAGGAAT GTGTAGGCTGGAGCTGCTTC
	R	TACAGAAATAGCTTACTTTCAGATAGTTCTAAAAGTAAGCTATGTTTTTA CATATGAATATCCTCCTTAG
sptP	F	TAACCCTGTTGAATGTTCCCACTCCCCTATTCAGGAATATTAAAAACGCT GTGTAGGCTGGAGCTGCTTC
	R	ACGCGTCATATAAACGATTTAATAGACTTTCCATATAGTTACCTCAAGAC CATATGAATATCCTCCTTAG
sopD	F	ATGCCAGTCACTTTAAGCTTCGGTAATCATCAAAATTA GTGTAGGCTGGAGCTGCTTC

	R	TTATGTCAGTAATATATTACGACTGCACCCATCTTTAC CATATGAATATCCTCCTTAG
avrA	F	ATGATATTTTCGGTGCAGGAGCTATCATGTGGAGGGAA GTGTAGGCTGGAGCTGCTTC
	R	TTAGCATAACGGCATTGTTATCGAATCGCTCATAAAGC CATATGAATATCCTCCTTAG
sopA	F	ATGAAGATATCATCAGGCGCAATTAATTTTTCTACTAT GTGTAGGCTGGAGCTGCTTC
	R	CTACGCCCAGGCCAGTGGCAGGATGGATGACAGAACAC CATATGAATATCCTCCTTAG
ptsG	F	ATGTTTAAGAATGCATTTGCTAACCTGCAAAAGGTCGG GTGTAGGCTGGAGCTGCTTC
	R	TTAGCTGTTACGGATGTACTCATCCATTTCCGTTTTCA CATATGAATATCCTCCTTAG
manXYZ	F	ATAATAAAGGAGGTAGCAAGTGACCATTGCTATTGTTA GTGTAGGCTGGAGCTGCTTC
	R	CGGCGATACCAATGACGAAGAAGCCCACGATAATCCAC CATATGAATATCCTCCTTAG
glk	F	ATGACAAAGTATGCTTTAGTAGGAGATGTAGGCGGCAC GTGTAGGCTGGAGCTGCTTC
	R	TTATAGAATATGACCTAATGTCTGGCGCAGATGCGCGC CATATGAATATCCTCCTTAG
creB-FLAG	F	ACACGCATCGTGGAATGGGATACAGCCTGAGGAGCGTC GACTACAAAGACCATGACGGT
	R	AAGAAAGTAGCCCAGCAGCAAACGCATTCCGATGCGCA TTACGCCCCGCCCTGCCACTC
vrpA-FLAG	F	TAACCCCGGAAGTACATTGATATTGTCTGGTAATATAT GACTACAAAGACCATGACGGT
	R	TAAAAACACGAGTGGATGAACAATCAATGCAAATAACC TTACGCCCCGCCCTGCCACTC
vrpB-FLAG	F	TGGCCCTGAGTCGCTTTTTGTATTTTTTAGCCCAGGTT GACTACAAAGACCATGACGGT
	R	GCAGGAACATCGGGCAATGCCCCTGGTCGGCGGCGCTA TTACGCCCCGCCCTGCCACTC
crp-FLAG	F	CCGCGCATGGCAAGACCATCGTCGTCTACGGCACCCGT GACTACAAAGACCATGACGGT
	R	AAAAATGGCGCATGATAAAACGCGCCATTCTGACGGAA TTACGCCCCGCCCTGCCACTC
Primers used for identification of the mutants		

pgtP	F	ACATTTATTACGCCAGAG
	R	TGCCAGTGAATAGATAGCG
creB	F	GGATTGGCTGAGACGAAA
	R	GCTGGTGATATGGGATAGTG
vrpA	F	ACACGAGTGGATGAACAATC
	R	TAAGGCGGCAACGATGTC
vrpB	F	GCAGGGAGATAGATGTTG
	R	AGTGAATACCACGACGAT
crp	F	AAGCGAGACACCAGGAGAC
	R	ACCAGCGTTTGCCGTAGT
cstA-ydbD	F	TGTAACATCTCTCTGGAACAC
	R	TGCGTTTATCAGGCCTGGCA
lldP	F	CCATTATGCGTGTGGTTCTCA
	R	ATCTCGTCTGACAGGCGTT
uhpT	F	GCGCTATTGCTGTTGCCATT
	R	ATAACTGTCGACGTTCTGGC
gtgE	F	CCACGTCATTTACATTTAGCCACCT
	R	ATCGCAAATTGCGTCGAATCTC
sipABCD	F	CAAACTCCTCACTAAGACCAAATAC
	R	GCAGCAAAAGCCAGACAGT

sopE2	F	TCGATCACCTTGCGCCGAA
	R	GCGGCATAACCTCTCCTGACA
slrP	F	AGCGTGTCATGGCAACAGATTAC
	R	ATCGGCTGGTGATTCAGGAAGT
sspH1	F	CCGGTAACTGTCAGATCAGGTC
	R	CGCTACGCCCTGACTGAAGAA
steA	F	CACCTGACGATATTGAAGCAGCATA
	R	CGCCGGAGGTACGTGGAATT
steB	F	GCAGATGTCAGTCTTGTAAGAACGT
	R	CTGGTTGGCAAGAGCGTGAAAT
sopB	F	TACACGGTGGCTCGGCATCTTA
	R	GTCAGGATGTCGTCAGGCAATG
sptP	F	CTGGCGGCAGATTATGGTGATT
	R	GCGCTTATAATGCCGGGACG
sopD	F	CCAACCATAAATGTGCTGTAT
	R	CTGAAATACCATCCGCTGTG
avrA	F	TTCAGGAAACATACGGTCAT
	R	GATGGACTCTTCACCGCTAT
sopA	F	AGAAACCTGCCAGATAACAT
	R	GACACTAAACTACGCTCCCT

ptsG	F	GGCACTTAGATGTCCTGTCC
	R	GTACAGGGAAACATCAACAAC
manXYZ	F	TTTCGTTGTAACCCTCATCTG
	R	GCACCAGGTCCGTGATAGTC
glk	F	CGCTCAGCAAGAGTAAAGTG
	R	GCAGTCGTTGCCAAAGAAAT
creB-FLAG	F	GATGATTATGTCGCCAAGC
	R	TTCCGAATCAAACAACACCT
vrpA-FLAG	F	TGTGGACTTTAGTGCGAATAT
	R	CGTATCAGTTCGTATGAAAAGG
vrpB-FLAG	F	CAGTATTGATAACGCCCTCG
	R	TAAAGCCCTTGATGAGATGG
crp-FLAG	F	AAATCACTCGTCAGGAAATCG
	R	GAAAAGCAAGCCCAGGTGTA
Primers used for construction of clone and the complemented strains		
vrpA	F	CATGCCATGGCCATGCCTGAAGATGGTTGCTGCCCAG
	R	CCGCTCGAGTATATTACCAGACAATATCAAT
vrpB	F	CGGGATCCATGGCGAACTGGGCGCAGAA
	R	CCGCTCGAGCTAAACCTGGGCTAAA
creB	F	CGGGATCCATGCAGCAACCGCAGGTCTG

	R	CCGCTCGAGTCAGACGCTCCTCAGGCTG
crp	F	CATGCCATGGCCATGGTGCTTGGCAAACCGCAAAC
	R	CCGCTCGAGACGGGTGCCGTAGACGACGAT
creBC-lux	F	CCGCTCGAGTGATCCATAAAATATCCCCA
	R	CGGGATCCACTAACCAGACCTGCGGTTG
Primers used for qRT-PCR		
β-actin	F	GGAGGGGGTTGAGGTGTT
	R	GTGTGCACTTTTATTGGTCTCAA
phdgh	F	AGCCTTGGATTGGTCTGGC
	R	AAGTTCACGTCTGCCTGCTTA
pfkfb3	F	GACGACCCTACTGTTGTGGC
	R	GGTCCTGCACTCTGTTCACC
slc2a1	F	GATTCGCCCATTCCTGTCTC
	R	AGGTCCTTCTCATGGTGTTTGT
16S rRNA	F	GAAAGCGTGGGGAGCAAAC
	R	ACATGCTCCACCGCTTGTG
ssrA	F	CTGGACCTCTTGCTGGCTGAT
	R	TGGCGTAAGTCGGTTAGTTCCT
ssrB	F	AAGTTCTGTTAGCGGCATTGC
	R	AGCAGTTGATGATTGGTCGTGT

ssaG	F	TGGATATGCTCTCCCACATGG
	R	CTGCTGTAAGGCAAATTGCG
sifA	F	ATGATGCCACCATTATTCTTCG
	R	CGTCATTTGTGGATGCGATT
pgtA	F	ATGGCGGTTGATGCGGTGAA
	R	ACCTGTAACGTCTGCTGGCAAT
pgtB	F	CAACGGCTTCGTCAGCTCAGTA
	R	AGCGGAGTGGATGCGAGGAA
pgtC	F	CGAGCTGGTGATGGCAACGA
	R	TTCCGCATTCGCCGTATCAAGT
pgtP	F	CGGCCTGCTGAGTAGCTGTAT
	R	ACCGCACGCCATGAAGACT
crp	F	GCAGTGCTGATCAAAGATGAAG
	R	TTGTAGGAAATTTCAGCGACCT
vrpA	F	AGATGGTTGCTGCCCAGAGAGG
	R	CCAGTTCCGCTTGCGTCAGATC
vrpB	F	GTGGATGTGGTGGTAGGGC
	R	TCAATACTGATGCGAATAGGG
creB	F	GACTGCCCGTAGCGATGA
	R	AGAGCGGCGTACCAAACC

Primers used for EMSAs and ChIP-qPCR		
P_{pgtC}	F	CGAAACGACTACAGCATGAGAA
	R	TATAGTGAGATGAACATCAGGAGGTT
P_{rpoS}	F	AGTCGCGTCCAACACACG
	R	TCGCTACTATGGGTAGCACC
P_{vrpA}	F	CAATCAGCCACTCGCCTTTC
	R	AGTTCCGCTTGCGTCAGAT
P_{vrpB}	F	AGCTCAATGACCCGCTTG
	R	TCGCTATAACCTCCAGGT
P_{ssrA}	F	CCTTGTCACAGGCGATTCTA
	R	CCATTGATATATAAGCTGCG
P_{ssrB}	F	GCAAGATTTTCCAATCACTG
	R	AATGAGGCCAGGGTAATAAG
P_{creD}	F	TACGCCTGAAAATGGCGTT
	R	CGTTCTCCTTTGCAATGC

[*] Primers were designed to carry extensions homologous to 38–40 bp (underlined) of the target gene; F, forward; R, reverse.

REFERENCES

1. Jiang L*, et al.* PagR mediates the precise regulation of *Salmonella* pathogenicity island 2 gene expression in response to magnesium and phosphate signals in *Salmonella* Typhimurium. *Cellular microbiology* **22**, e13125 (2020).

2. Jiang L*, et al.* Signal transduction pathway mediated by the novel regulator LoiA for low oxygen tension induced *Salmonella* Typhimurium invasion. *PLoS pathogens* **13**, e1006429 (2017).
