# Supplementary material for: Salmonella Typhimurium reprograms macrophage metabolism via T3SS effector SopE2 to promote intracellular replication and virulence
Source: Nat Commun. 2021 Feb 9;12:879. doi: 10.1038/s41467-021-21186-4 (PMC7873081; doi:10.1038/s41467-021-21186-4)
Supplement: Supplementary file 3 — Reporting Summary [file 41467_2021_21186_MOESM3_ESM.pdf]

nature research

Corresponding author(s): Lei Wang

Last updated by author(s): Jan 6, 2021

Reporting Summary

Nature Research wishes to improve the reproducibility of the work that we publish. This form provides structure for consistency and transparency in reporting. For further information on Nature Research policies, see Authors & Referees and the Editorial Policy Checklist .

Statistics

For all statistical analyses, confirm that the following items are present in the figure legend, table legend, main text, or Methods section.

n/a	Confirmed	
☐	☒	The exact sample size (*n*) for each experimental group/condition, given as a discrete number and unit of measurement
☐	☒	A statement on whether measurements were taken from distinct samples or whether the same sample was measured repeatedly
☐	☒	The statistical test(s) used AND whether they are one- or two-sided<br>*Only common tests should be described solely by name; describe more complex techniques in the Methods section.*
☒	☐	A description of all covariates tested
☐	☒	A description of any assumptions or corrections, such as tests of normality and adjustment for multiple comparisons
☐	☒	A full description of the statistical parameters including central tendency (e.g. means) or other basic estimates (e.g. regression coefficient) AND variation (e.g. standard deviation) or associated estimates of uncertainty (e.g. confidence intervals)
☐	☒	For null hypothesis testing, the test statistic (e.g. *F*, *t*, *r*) with confidence intervals, effect sizes, degrees of freedom and *P* value noted<br>*Give P values as exact values whenever suitable.*
☒	☐	For Bayesian analysis, information on the choice of priors and Markov chain Monte Carlo settings
☒	☐	For hierarchical and complex designs, identification of the appropriate level for tests and full reporting of outcomes
☒	☐	Estimates of effect sizes (e.g. Cohen's *d*, Pearson's *r*), indicating how they were calculated

Our web collection on statistics for biologists contains articles on many of the points above.

Software and code

Policy information about availability of computer code

Data collection	Sequencing data was collected by Illumina HiSeq 2500 (Illumina).
Data analysis	GraphPad Prism 8.0.1; Image J180; ZEN 2.3 (blue edition); Xcalibur 4.0; Bowtie (version 2.2.3); HTSeq (version 0.6.1); DESeq2; BWA mem (version 0.7.12); Cutadapt (v1.9.1); MACS2 (v2.1.0)

For manuscripts utilizing custom algorithms or software that are central to the research but not yet described in published literature, software must be made available to editors/reviewers. We strongly encourage code deposition in a community repository (e.g. GitHub). See the Nature Research guidelines for submitting code & software for further information.

Data

Policy information about availability of data

All manuscripts must include a data availability statement. This statement should provide the following information, where applicable:

- Accession codes, unique identifiers, or web links for publicly available datasets
- A list of figures that have associated raw data
- A description of any restrictions on data availability

The RNA-seq and ChIP-seq data acquired in this study are available in the NCBI Sequence Read Archive (SRA, PRJNA561041, https://www.ncbi.nlm.nih.gov/sra/PRJNA561041). The metabolomic data have been deposited in MetaboLights (MTBLS2347, www.ebi.ac.uk/metabolights/MTBLS2347). Source data are provided with this paper.

Field-specific reporting

Please select the one below that is the best fit for your research. If you are not sure, read the appropriate sections before making your selection.

☒

October 2018

Life sciences study design

All studies must disclose on these points even when the disclosure is negative.

Sample size	ChIP-seq was performed once for the ChIP sample and the mock ChIP sample. All other in vitro experiments were performed in duplicate and repeated at least three times (n ≥ 3). Mouse virulence assays were conducted twice with at least 3 mouse (n ≥ 3) in each injection group, and the combined data for the two experiments was used for statistical analysis.
Data exclusions	No data were excluded from the analyses.
Replication	All the reported experiments were reproducible. Data reproducibility was confirmed by two or three independent experiments. ChIP-seq results were validated by three independent ChIP-qPCR analyses on target genes.
Randomization	Mice were randomized into the different groups with same gender and similar weight.
Blinding	Sequencing of Tn libraries and cDNA libraries was done blindly by Majorbio Bio-Pharm Technology Co., Ltd. (Shanghai, China), which also provided the statistical analysis. Other experiments were not done blindly. Most experiments were conducted by at least two different researchers who have not known the situation and results of the study in advance and repeated on at least two independent days.

Reporting for specific materials, systems and methods

We require information from authors about some types of materials, experimental systems and methods used in many studies. Here, indicate whether each material, system or method listed is relevant to your study. If you are not sure if a list item applies to your research, read the appropriate section before selecting a response.

Materials & experimental systems

n/a	Involved in the study	
☐	☒	Antibodies
☐	☒	Eukaryotic cell lines
☒	☐	Palaeontology
☐	☒	Animals and other organisms
☒	☐	Human research participants
☒	☐	Clinical data

Methods

n/a	Involved in the study	
☐	☒	ChIP-seq
☒	☐	Flow cytometry
☒	☐	MRI-based neuroimaging

Antibodies

Antibodies used	Anti-Actin antibody cat.ab179467. Abcam. 1:2,000-1:5,000 dilution used for immunoblotting.<br>Anti-PHGDH antibody cat.13428. Cell Signaling Technology. 1:1,000 dilution used for immunoblotting.<br>Anti-PFKFB3 antibody cat.ab181861. Abcam. 1:1,000 dilution used for immunoblotting.<br>Anti-CDC42 antibody cat.ab187643. Abcam. 1:1,000 dilution used for immunoblotting.<br>HRP-conjugated goat anti-rabbit IgG secondary antibody cat.EF0002. Sparkjade. 1:5,000 dilution used for immunoblotting.<br>Anti-Salmonella typhimurium LPS antibody cat.ab8274. Abcam. 1:100 dilution used for immunofluorescence.<br>Goat anti-mouse IgG (FITC) cat.ab6785. Abcam. 1:200 dilution used for immunofluorescence.<br>Anti-FLAG mouse monoclonal antibody cat.F1804. Sigma. 1:1,000 dilution used for chromatin immunoprecipitation.
Validation	All antibodies were obtained commercially and were tested and validated by the respective company. All antibodies had validation statement provided on the website of the manufacturer.<br>Anti-Actin antibody: rabbit monoclonal to Actin; suitable for WB, IHC-P, ICC/IF, IP, and Flow Cyt; reacts with mouse, rat, chicken, and human.<br>Anti-PHGDH antibody: rabbit polyclonal to PHGDH; suitable for WB; reacts with mouse, rat, and human.<br>Anti-PFKFB3 antibody: rabbit monoclonal to PFKFB3; suitable for WB, IHC-P, ICC/IF, IP, and Flow Cyt; reacts with mouse, rat, and human.<br>Anti-CDC42 antibody: rabbit monoclonal to CDC42; suitable for WB, IHC-P, ICC/IF, IP, and Flow Cyt; reacts with mouse, rat, and human.<br>Anti-Salmonella typhimurium LPS antibody: mouse monoclonal to Salmonella typhimurium LPS; suitable for WB, Dot blot, ELISA, ICC/IF, and IHC-Fr; reacts with Salmonella typhimurium.<br>Anti-FLAG mouse monoclonal antibody: mouse monoclonal to FLAG-tag; suitable for WB, IP, ICC/IF, and IHC-Fr; reacts with FLAG-tag (DYKDDDDK).

Eukaryotic cell lines

Policy information about cell lines

Cell line source(s)	The RAW264.7 macrophage-like cell line (ATCC TIB-71) and HeLa cell line (ATCC CCL-2) were purchased from the Shanghai Institute of Biochemistry and Cell Biology of the Chinese Academy of Sciences (Shanghai, China).
Authentication	All the cell lines of the Shanghai Institute of Biochemistry and Cell Biology are originated from ATCC. ATCC authenticates its cell lines through morphology, karyotyping, and STR analyses, thus RAW264.7 and HeLa cells were not authenticated after receipt. Regular inspection of cell culture for coherent morphology with ATCC source images was routinely performed.
Mycoplasma contamination	Cells routinely tested negative for mycoplasma contamination.
Commonly misidentified lines (See ICLAC register)	No commonly misidentified cell lines were used.

Animals and other organisms

Policy information about studies involving animals; ARRIVE guidelines recommended for reporting animal research

Laboratory animals	Male BALB/c mice (six to eight weeks old) purchased from Beijing Vital River Laboratory Animal Technology (Beijing, China) were used for all animal experiments in this work. Mice were housed under specific pathogen-free conditions with a 12 h light/dark cycle, at a temperature of 22 ± 2°C, and a relative humidity of 50% ± 5%. Mice were fed a standard mouse chow diet.
Wild animals	No wild animals were used in the study.
Field-collected samples	No field-collected samples were employed in this study.
Ethics oversight	All animal studies were conducted according to protocols approved by the Institutional Animal Care Committee of Nankai University (Tianjin, China) and performed under protocol no. IACUC 2016030502.

Note that full information on the approval of the study protocol must also be provided in the manuscript.

ChIP-seq

Data deposition

- [x] Confirm that both raw and final processed data have been deposited in a public database such as GEO.
- [x] Confirm that you have deposited or provided access to graph files (e.g. BED files) for the called peaks.

Data access links *May remain private before publication.*	https://www.ncbi.nlm.nih.gov/sra/PRJNA561041
Files in database submission	creB_0_1.fq.gz, creB_IP_1.fq.gz
Genome browser session (e.g. UCSC)	ftp://ftp.ncbi.nlm.nih.gov/genomes/archive/old_genbank/Bacteria<br>Salmonella_enterica_serovar_Typhimurium_14028S_uid33067/

Methodology

Replicates	ChIP-seq was performed once for the ChIP sample (creB_IP) and the mock ChIP sample (creB_0). The promoter region of vrpB was significantly enriched in the ChIP sample compared to that in the mock ChIP sample. The ChIP-qPCR results (from three independent biological experiments) confirmed the enrichment of the vrpB promoter region in the ChIP sample (Fig. 5g).
Sequencing depth	Sequencing layout: 2×150 paired-end<br>Sequencing Depth for each sample (ID: total number of reads/uniquely mapped)<br>creB_0.fq.gz: 22,333,649/21,449,212<br>creB_IP.fq.gz: 18,352,530/17,232,568
Antibodies	Anti-FLAG mouse monoclonal antibody (Sigma #F1804)
Peak calling parameters	Peaks were called using MACS2 with default settings and the fragment size set to 150 bp (option '--extsize 150').
Data quality	Only uniquely mapped reads with an alignment score ≥ 20 were used for peak calling. After mapping reads to the reference genome, the MACS2 (v2.1.0) peak finding algorithm was used to identify regions of IP enrichment over control. A q value threshold of enrichment of 0.05 was used for all data sets. Number of peaks: 773
Software	Quality control: Cutadapt (v1.9.1)<br>Read mapping: BWA mem (v0.7.12)

Peak calling: MACS2 (v2.1.0)
